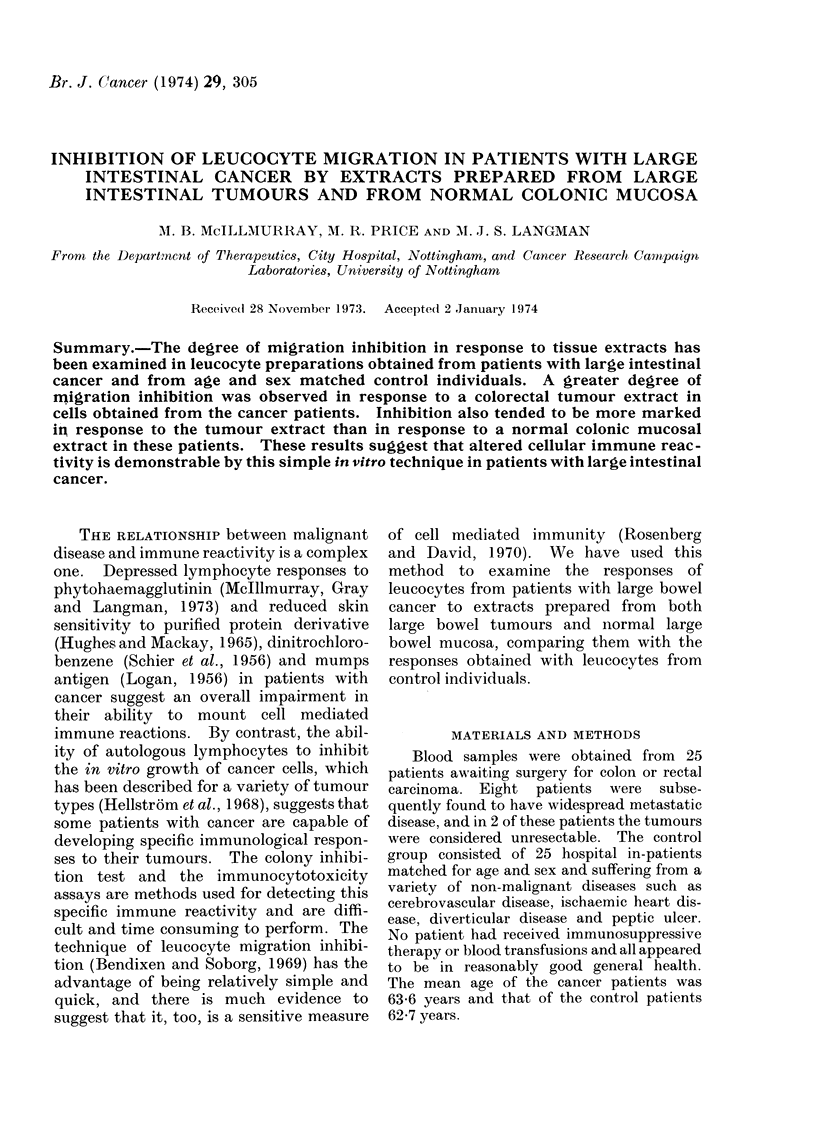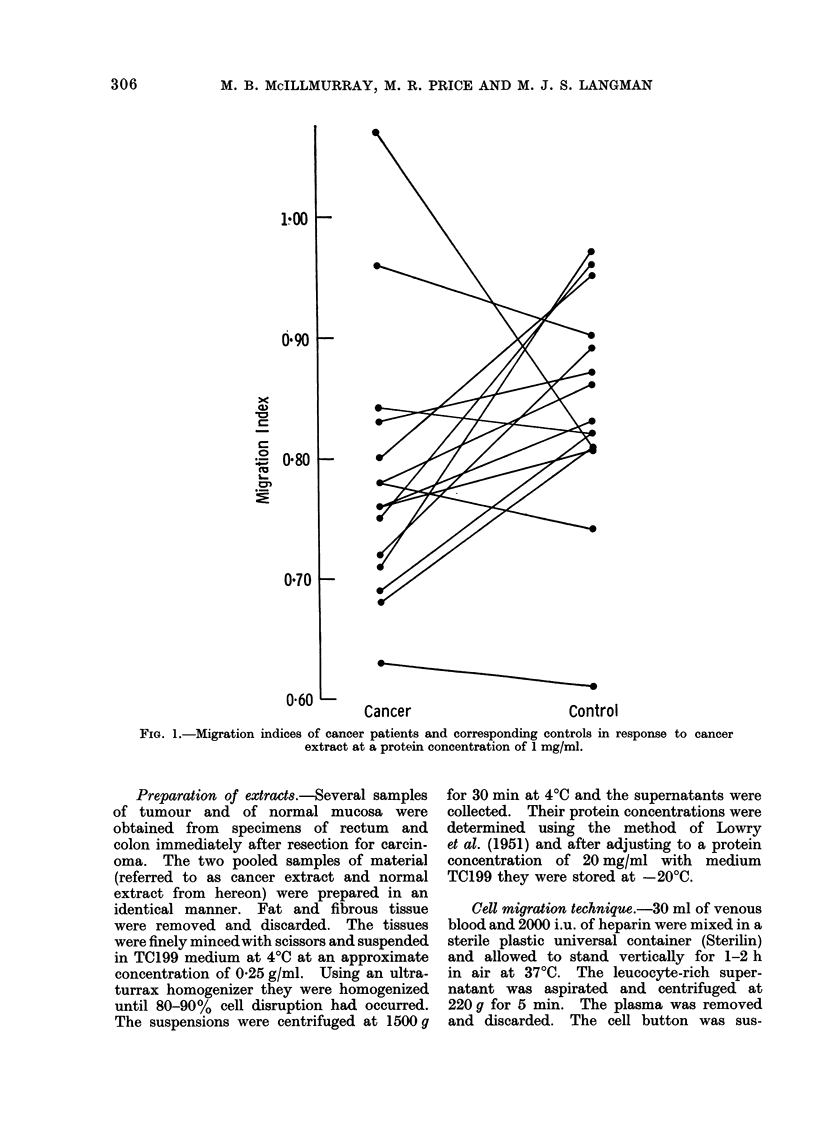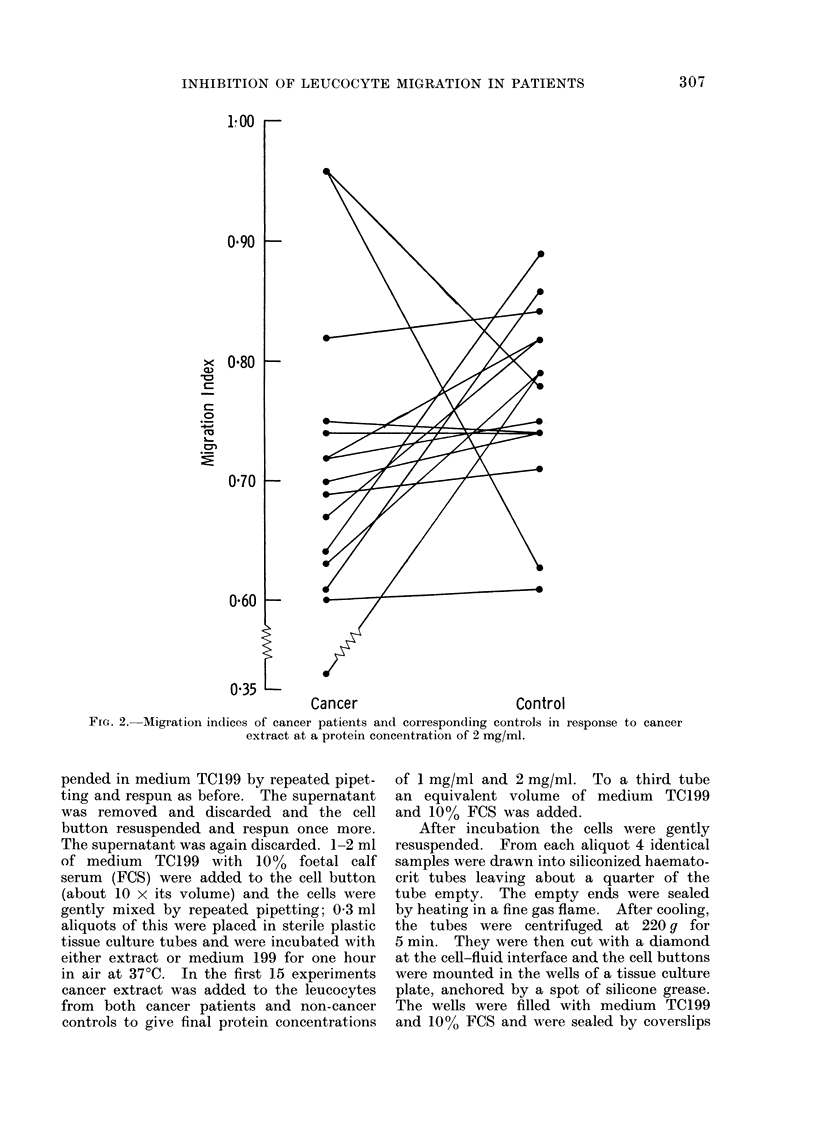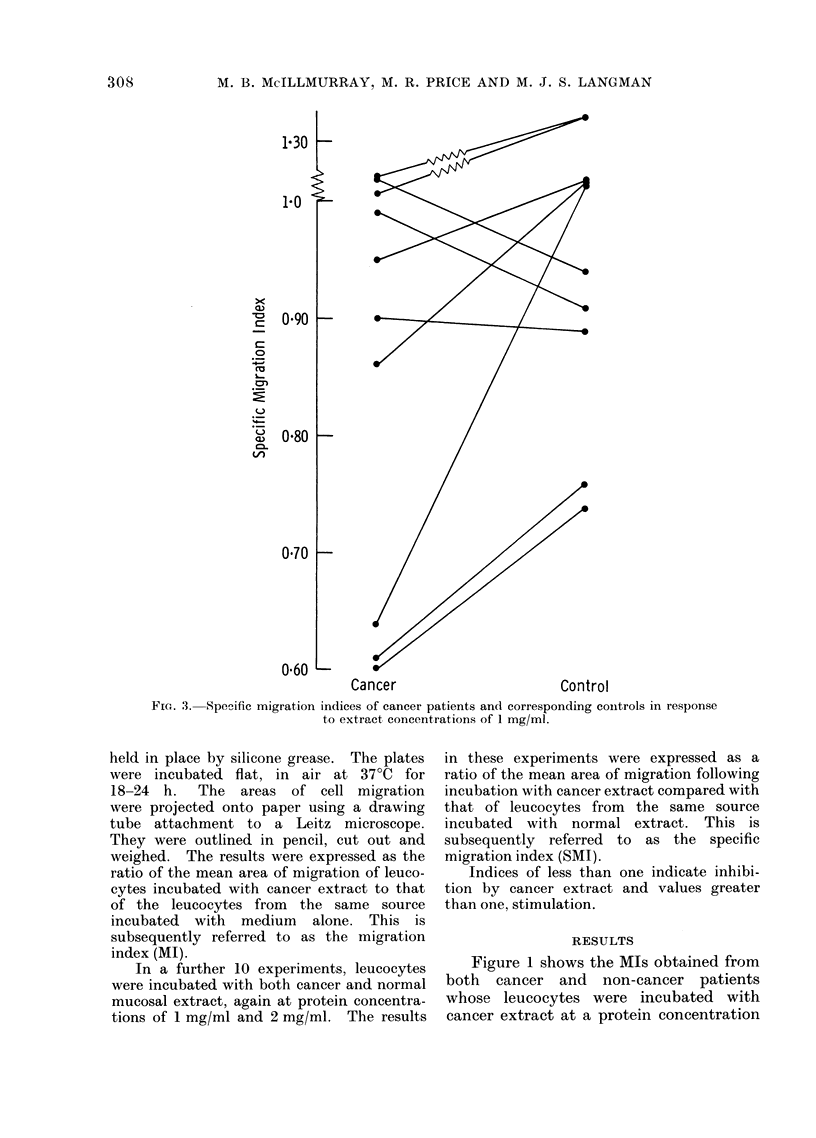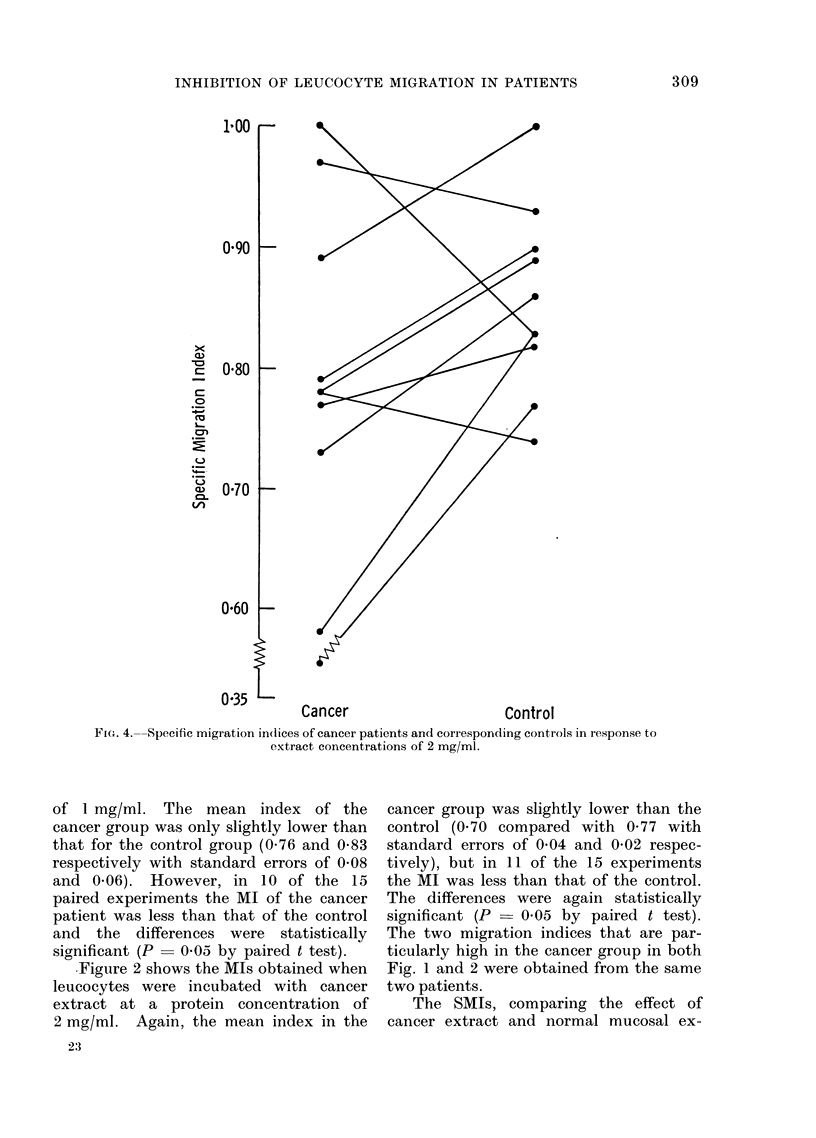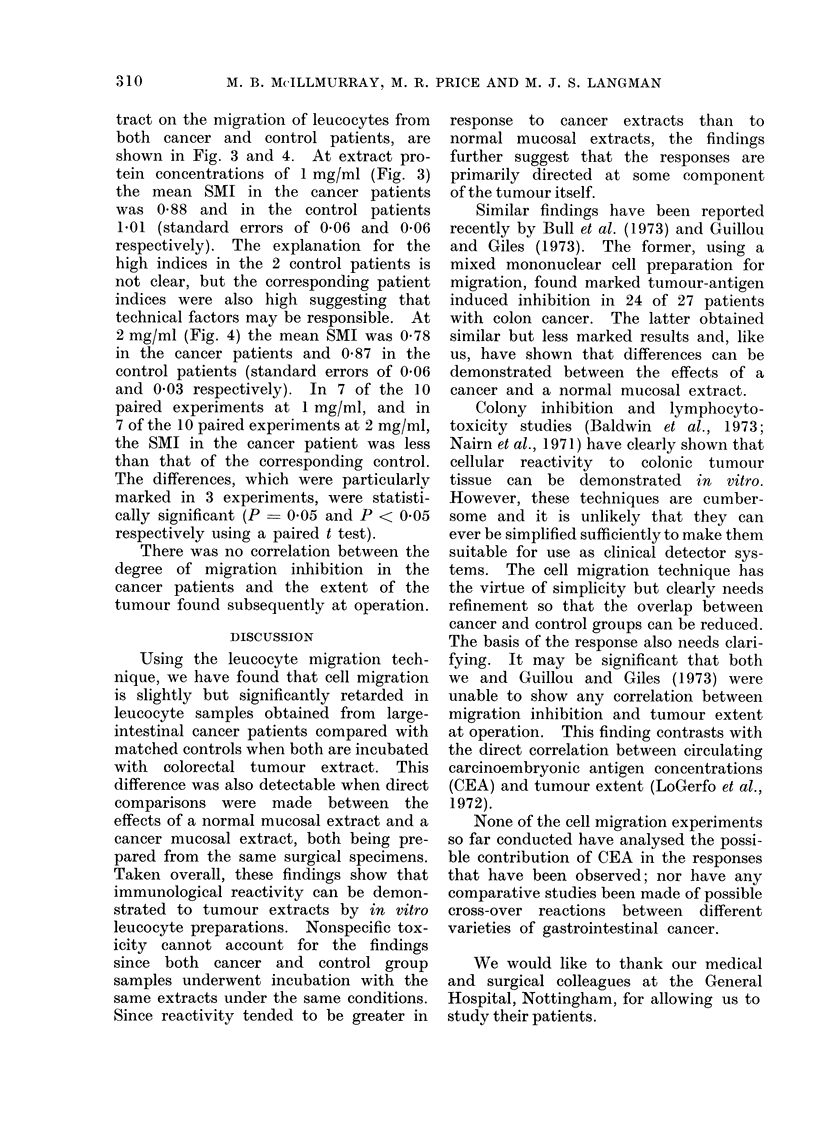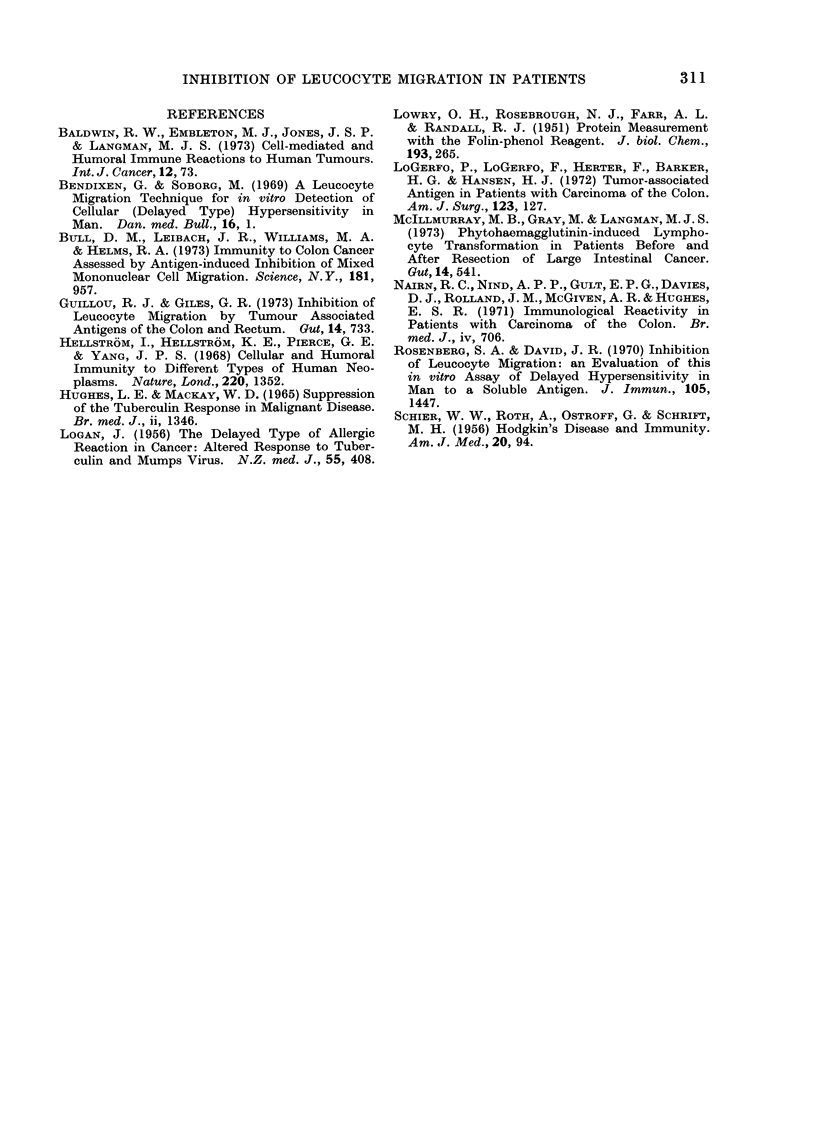# Inhibition of Leucocyte Migration in Patients with Large Intestinal Cancer by Extracts Prepared from Large Intestinal Tumours and from Normal Colonic Mucosa

**DOI:** 10.1038/bjc.1974.71

**Published:** 1974-04

**Authors:** M. B. McIllmurray, M. R. Price, M. J. S. Langman

## Abstract

The degree of migration inhibition in response to tissue extracts has been examined in leucocyte preparations obtained from patients with large intestinal cancer and from age and sex matched control individuals. A greater degree of migration inhibition was observed in response to a colorectal tumour extract in cells obtained from the cancer patients. Inhibition also tended to be more marked in response to the tumour extract than in response to a normal colonic mucosal extract in these patients. These results suggest that altered cellular immune reactivity is demonstrable by this simple *in vitro* technique in patients with large intestinal cancer.


*Br. J. Cancer* (1974) **29**, 305

### INHIBITION OF LEUCOCYTE MIGRATION IN PATIENTS WITH LARGE INTESTINAL CANCER BY EXTRACTS PREPARED FROM LARGE INTESTINAL TUMOURS AND FROM NORMAL COLONIC MUCOSA

M. B. McILLMURRAY, M. R. PRICE AND M. J. S. LANGMAN

*From the Department of Therapeutics, City Hospital, Nottingham, and Cancer Research Campaign Laboratories, University of Nottingham*

Received 28 November 1973. Accepted 2 January 1974

**Summary.—The degree of migration inhibition in response to tissue extracts has been examined in leucocyte preparations obtained from patients with large intestinal cancer and from age and sex matched control individuals. A greater degree of migration inhibition was observed in response to a colorectal tumour extract in cells obtained from the cancer patients. Inhibition also tended to be more marked in response to the tumour extract than in response to a normal colonic mucosal extract in these patients. These results suggest that altered cellular immune reactivity is demonstrable by this simple *in vitro* technique in patients with large intestinal cancer.**

THE RELATIONSHIP between malignant disease and immune reactivity is a complex one. Depressed lymphocyte responses to phytohaemagglutinin (McIllmurray, Gray and Langman, 1973) and reduced skin sensitivity to purified protein derivative (Hughes and Mackay, 1965), dinitrochlorobenzene (Schier *et al.*, 1956) and mumps antigen (Logan, 1956) in patients with cancer suggest an overall impairment in their ability to mount cell mediated immune reactions. By contrast, the ability of autologous lymphocytes to inhibit the *in vitro* growth of cancer cells, which has been described for a variety of tumour types (Hellström *et al.*, 1968), suggests that some patients with cancer are capable of developing specific immunological responses to their tumours. The colony inhibition test and the immunocytotoxicity assays are methods used for detecting this specific immune reactivity and are difficult and time consuming to perform. The technique of leucocyte migration inhibition (Bendixen and Soborg, 1969) has the advantage of being relatively simple and quick, and there is much evidence to suggest that it, too, is a sensitive measure of cell mediated immunity (Rosenberg and David, 1970). We have used this method to examine the responses of leucocytes from patients with large bowel cancer to extracts prepared from both large bowel tumours and normal large bowel mucosa, comparing them with the responses obtained with leucocytes from control individuals.

### MATERIALS AND METHODS

Blood samples were obtained from 25 patients awaiting surgery for colon or rectal carcinoma. Eight patients were subsequently found to have widespread metastatic disease, and in 2 of these patients the tumours were considered unresectable. The control group consisted of 25 hospital in-patients matched for age and sex and suffering from a variety of non-malignant diseases such as cerebrovascular disease, ischaemic heart disease, diverticular disease and peptic ulcer. No patient had received immunosuppressive therapy or blood transfusions and all appeared to be in reasonably good general health. The mean age of the cancer patients was 63·6 years and that of the control patients 62·7 years.

FIG. 1.—Migration indices of cancer patients and corresponding controls in response to cancer extract at a protein concentration of 1 mg/ml.

*Preparation of extracts.*—Several samples of tumour and of normal mucosa were obtained from specimens of rectum and colon immediately after resection for carcinoma. The two pooled samples of material (referred to as cancer extract and normal extract from hereon) were prepared in an identical manner. Fat and fibrous tissue were removed and discarded. The tissues were finely minced with scissors and suspended in TC199 medium at 4°C at an approximate concentration of 0·25 g/ml. Using an ultra-turrax homogenizer they were homogenized until 80–90% cell disruption had occurred. The suspensions were centrifuged at 1500 *g* for 30 min at 4°C and the supernatants were collected. Their protein concentrations were determined using the method of Lowry *et al.* (1951) and after adjusting to a protein concentration of 20 mg/ml with medium TC199 they were stored at −20°C.

*Cell migration technique.*—30 ml of venous blood and 2000 i.u. of heparin were mixed in a sterile plastic universal container (Sterilin) and allowed to stand vertically for 1–2 h in air at 37°C. The leucocyte-rich supernatant was aspirated and centrifuged at 220 *g* for 5 min. The plasma was removed and discarded. The cell button was sus-

FIG. 2.—Migration indices of cancer patients and corresponding controls in response to cancer extract at a protein concentration of 2 mg/ml.

pended in medium TC199 by repeated pipetting and respun as before. The supernatant was removed and discarded and the cell button resuspended and respun once more. The supernatant was again discarded. 1–2 ml of medium TC199 with 10% foetal calf serum (FCS) were added to the cell button (about 10 × its volume) and the cells were gently mixed by repeated pipetting; 0·3 ml aliquots of this were placed in sterile plastic tissue culture tubes and were incubated with either extract or medium 199 for one hour in air at 37°C. In the first 15 experiments cancer extract was added to the leucocytes from both cancer patients and non-cancer controls to give final protein concentrations of 1 mg/ml and 2 mg/ml. To a third tube an equivalent volume of medium TC199 and 10% FCS was added.

After incubation the cells were gently resuspended. From each aliquot 4 identical samples were drawn into siliconized haematocrit tubes leaving about a quarter of the tube empty. The empty ends were sealed by heating in a fine gas flame. After cooling, the tubes were centrifuged at 220 *g* for 5 min. They were then cut with a diamond at the cell–fluid interface and the cell buttons were mounted in the wells of a tissue culture plate, anchored by a spot of silicone grease. The wells were filled with medium TC199 and 10% FCS and were sealed by coverslips

FIG. 3.—Specific migration indices of cancer patients and corresponding controls in response to extract concentrations of 1 mg/ml.

held in place by silicone grease. The plates were incubated flat, in air at 37°C for 18–24 h. The areas of cell migration were projected onto paper using a drawing tube attachment to a Leitz microscope. They were outlined in pencil, cut out and weighed. The results were expressed as the ratio of the mean area of migration of leucocytes incubated with cancer extract to that of the leucocytes from the same source incubated with medium alone. This is subsequently referred to as the migration index (MI).

In a further 10 experiments, leucocytes were incubated with both cancer and normal mucosal extract, again at protein concentrations of 1 mg/ml and 2 mg/ml. The results in these experiments were expressed as a ratio of the mean area of migration following incubation with cancer extract compared with that of leucocytes from the same source incubated with normal extract. This is subsequently referred to as the specific migration index (SMI).

Indices of less than one indicate inhibition by cancer extract and values greater than one, stimulation.

### RESULTS

Figure 1 shows the MIs obtained from both cancer and non-cancer patients whose leucocytes were incubated with cancer extract at a protein concentration

FIG. 4.—Specific migration indices of cancer patients and corresponding controls in response to extract concentrations of 2 mg/ml.

of 1 mg/ml. The mean index of the cancer group was only slightly lower than that for the control group (0·76 and 0·83 respectively with standard errors of 0·08 and 0·06). However, in 10 of the 15 paired experiments the MI of the cancer patient was less than that of the control and the differences were statistically significant ($P = 0{\cdot}05$ by paired $t$ test).

Figure 2 shows the MIs obtained when leucocytes were incubated with cancer extract at a protein concentration of 2 mg/ml. Again, the mean index in the cancer group was slightly lower than the control (0·70 compared with 0·77 with standard errors of 0·04 and 0·02 respectively), but in 11 of the 15 experiments the MI was less than that of the control. The differences were again statistically significant ($P = 0{\cdot}05$ by paired $t$ test). The two migration indices that are particularly high in the cancer group in both Fig. 1 and 2 were obtained from the same two patients.

The SMIs, comparing the effect of cancer extract and normal mucosal extract on the migration of leucocytes from both cancer and control patients, are shown in Fig. 3 and 4. At extract protein concentrations of 1 mg/ml (Fig. 3) the mean SMI in the cancer patients was 0·88 and in the control patients 1·01 (standard errors of 0·06 and 0·06 respectively). The explanation for the high indices in the 2 control patients is not clear, but the corresponding patient indices were also high suggesting that technical factors may be responsible. At 2 mg/ml (Fig. 4) the mean SMI was 0·78 in the cancer patients and 0·87 in the control patients (standard errors of 0·06 and 0·03 respectively). In 7 of the 10 paired experiments at 1 mg/ml, and in 7 of the 10 paired experiments at 2 mg/ml, the SMI in the cancer patient was less than that of the corresponding control. The differences, which were particularly marked in 3 experiments, were statistically significant ($P = 0{\cdot}05$ and $P < 0{\cdot}05$ respectively using a paired *t* test).

There was no correlation between the degree of migration inhibition in the cancer patients and the extent of the tumour found subsequently at operation.

### DISCUSSION

Using the leucocyte migration technique, we have found that cell migration is slightly but significantly retarded in leucocyte samples obtained from large-intestinal cancer patients compared with matched controls when both are incubated with colorectal tumour extract. This difference was also detectable when direct comparisons were made between the effects of a normal mucosal extract and a cancer mucosal extract, both being prepared from the same surgical specimens. Taken overall, these findings show that immunological reactivity can be demonstrated to tumour extracts by *in vitro* leucocyte preparations. Nonspecific toxicity cannot account for the findings since both cancer and control group samples underwent incubation with the same extracts under the same conditions. Since reactivity tended to be greater in response to cancer extracts than to normal mucosal extracts, the findings further suggest that the responses are primarily directed at some component of the tumour itself.

Similar findings have been reported recently by Bull *et al.* (1973) and Guillou and Giles (1973). The former, using a mixed mononuclear cell preparation for migration, found marked tumour-antigen induced inhibition in 24 of 27 patients with colon cancer. The latter obtained similar but less marked results and, like us, have shown that differences can be demonstrated between the effects of a cancer and a normal mucosal extract.

Colony inhibition and lymphocytotoxicity studies (Baldwin *et al.*, 1973; Nairn *et al.*, 1971) have clearly shown that cellular reactivity to colonic tumour tissue can be demonstrated *in vitro*. However, these techniques are cumbersome and it is unlikely that they can ever be simplified sufficiently to make them suitable for use as clinical detector systems. The cell migration technique has the virtue of simplicity but clearly needs refinement so that the overlap between cancer and control groups can be reduced. The basis of the response also needs clarifying. It may be significant that both we and Guillou and Giles (1973) were unable to show any correlation between migration inhibition and tumour extent at operation. This finding contrasts with the direct correlation between circulating carcinoembryonic antigen concentrations (CEA) and tumour extent (LoGerfo *et al.*, 1972).

None of the cell migration experiments so far conducted have analysed the possible contribution of CEA in the responses that have been observed; nor have any comparative studies been made of possible cross-over reactions between different varieties of gastrointestinal cancer.

We would like to thank our medical and surgical colleagues at the General Hospital, Nottingham, for allowing us to study their patients.

### REFERENCES

Baldwin, R. W., Embleton, M. J., Jones, J. S. P. & Langman, M. J. S. (1973) Cell-mediated and Humoral Immune Reactions to Human Tumours. *Int. J. Cancer*, **12**, 73.

Bendixen, G. & Soborg, M. (1969) A Leucocyte Migration Technique for *in vitro* Detection of Cellular (Delayed Type) Hypersensitivity in Man. *Dan. med. Bull.*, **16**, 1.

Bull, D. M., Leibach, J. R., Williams, M. A. & Helms, R. A. (1973) Immunity to Colon Cancer Assessed by Antigen-induced Inhibition of Mixed Mononuclear Cell Migration. *Science, N.Y.*, **181**, 957.

Guillou, R. J. & Giles, G. R. (1973) Inhibition of Leucocyte Migration by Tumour Associated Antigens of the Colon and Rectum. *Gut*, **14**, 733.

Hellström, I., Hellström, K. E., Pierce, G. E. & Yang, J. P. S. (1968) Cellular and Humoral Immunity to Different Types of Human Neoplasms. *Nature, Lond.*, **220**, 1352.

Hughes, L. E. & Mackay, W. D. (1965) Suppression of the Tuberculin Response in Malignant Disease. *Br. med. J.*, ii, 1346.

Logan, J. (1956) The Delayed Type of Allergic Reaction in Cancer: Altered Response to Tuberculin and Mumps Virus. *N.Z. med. J.*, **55**, 408.

Lowry, O. H., Rosebrough, N. J., Farr, A. L. & Randall, R. J. (1951) Protein Measurement with the Folin-phenol Reagent. *J. biol. Chem.*, **193**, 265.

LoGerfo, P., LoGerfo, F., Herter, F., Barker, H. G. & Hansen, H. J. (1972) Tumor-associated Antigen in Patients with Carcinoma of the Colon. *Am. J. Surg.*, **123**, 127.

McIllmurray, M. B., Gray, M. & Langman, M. J. S. (1973) Phytohaemagglutinin-induced Lymphocyte Transformation in Patients Before and After Resection of Large Intestinal Cancer. *Gut*, **14**, 541.

Nairn, R. C., Nind, A. P. P., Gult, E. P. G., Davies, D. J., Rolland, J. M., McGiven, A. R. & Hughes, E. S. R. (1971) Immunological Reactivity in Patients with Carcinoma of the Colon. *Br. med. J.*, iv, 706.

Rosenberg, S. A. & David, J. R. (1970) Inhibition of Leucocyte Migration: an Evaluation of this *in vitro* Assay of Delayed Hypersensitivity in Man to a Soluble Antigen. *J. Immun.*, **105**, 1447.

Schier, W. W., Roth, A., Ostroff, G. & Schrift, M. H. (1956) Hodgkin's Disease and Immunity. *Am. J. Med.*, **20**, 94.